# Ketamine and its combinations with valproate and carbamazepine are ineffective against convulsions induced by atropine treatment and food intake in fasted mice

**DOI:** 10.22038/ijbms.2019.33890.8062

**Published:** 2019-03

**Authors:** Neriman Gözüaçık, Aslı Zengin Türkmen, Asiye Nurten, Nurhan Enginar

**Affiliations:** 1 Department of Medical Pharmacology, Istanbul Faculty of Medicine, Istanbul University, Istanbul, Turkey; 2 Department of Physiology, Faculty of Medicine, Istanbul Yeni Yuzyil University, Istanbul, Turkey

**Keywords:** Atropine, Carbamazepine, Convulsion, Fasting, Glutamate, Ketamine, Valproate

## Abstract

**Objective(s):**

Fasted rodents treated with antimuscarinics develop convulsions after refeeding. Food deprivation for 48 hr produces changes in [3H]glutamate binding suggesting glutamatergic contribution to the underlying mechanism of the seizures that are somewhat unresponsive to antiepileptics. Studies in animals and epileptic patients yielded considerable information regarding the anticonvulsant effect of the noncompetitive N-methyl-D-aspartate (NMDA) receptor antagonist ketamine. Thus, this study evaluated the efficacy of ketamine and its combinations with valproate and carbamazepine on convulsions in fasted animals.

**Materials and Methods:**

Following 24 hr of fasting, mice were given saline, 5 or 10 mg/kg ketamine, 250 mg/kg sodium valproate, 24 mg/kg carbamazepine, 5 mg/kg ketamine+sodium valproate, or 5 mg/kg ketamine+carbamazepine and then were treated with saline or 2.4 mg/kg atropine (5-9 mice per group). The animals were observed for the occurrence of convulsions after being allowed to eat *ad libitum.*

**Results:**

Ketamine, valproate and carbamazepine pretreatments were ineffective in preventing the convulsions developed after atropine treatment and food intake in fasted animals. The incidence of convulsions was significantly higher in 5 and 10 mg/kg ketamine, carbamazepine, and carbamazepine+ketamine groups, but not in the valproate and valproate+ketamine treated animals.

**Conclusion:**

In contrast to previous findings obtained with the NMDA antagonist dizocilpine (MK-801), ketamine lacks activity against convulsions developed after fasting. The drug does not enhance the efficacy of valproate and carbamazepine either. Using different doses of ketamine or other NMDA antagonists, further studies may better clarify the anticonvulsant effect of ketamine and/or role of glutamate in these seizures.

## Introduction

Fasted mice and rats treated with muscarinic antagonists, scopolamine, atropine, biperiden, or pirenzepine develop convulsions soon after food intake (1, 2). Food deprivation itself, rather than its hypoglycemic consequence, seems to be critical in the occurrence of these seizures. Deprivation of food for 48 hr produced significant changes in the kinetics of (^3^H)glutamate binding in the brain, which were partly antagonized by scopolamine treatment and food intake (3). Fasting increased expression of M_1_ muscarinic receptors in the frontal cortex and M_2_ muscarinic receptors in the hippocampus (2). Sight or smell of food, chewing, swallowing or the complete act of eating a meal may act as triggering factors (4). Dopaminergic D_2_ receptor antagonists chlorpromazine and haloperidol, glutamatergic N-methyl-D-aspartate (NMDA) receptor antagonist MK-801 and alpha-2 adrenergic receptor agonists clonidine and tizanidine provided effective treatments. However, convulsions are somewhat unresponsive to most of the antiepileptic drugs (5, 6). Bearing some similarities in triggering factors and manifestations of the seizures and response to therapy in patients with eating epilepsy, convulsions in fasted animals may provide insight into the unknown underlying mechanism(s) of this rare form of reflex seizures (7, 8, 9).

The anesthetic drug ketamine is a glutamatergic noncompetitive NMDA receptor antagonist. It has potent analgesic and sedative effects (10). Animal studies have shown that ketamine exerts anticonvulsant effects in a variety of seizure models (11). The combinations of ketamine and conventional antiepileptics reduce seizure scores at non-effective doses (11, 12). Clinically, ketamine is used in the treatment of patients with status epilepticus refractory to benzodiazepines, barbiturates, and diphenylhydantoin (13). However, a proconvulsant effect for ketamine has also been documented (14, 15).

In view of the findings mentioned above, the present study was performed to evaluate the efficacy of ketamine and its combinations with valproate and carbamazepine on convulsions induced by antimuscarinic treatment and food intake after fasting. Atropine was used as the antimuscarinic drug (16).

## Materials and Methods

***Animals***

Inbred male BALB/c mice weighing 25–35 g were housed under standard laboratory conditions for at least 1 week prior to experimentation and were allowed free access to both food and water. All studies were approved by the Istanbul University Local Ethics Committee on Animal Experiments (2012/181) and were in accordance with the *EU Directive 2010/63/EU* on the protection of animals used for scientific purposes.

***Drugs***

Atropine sulfate (Sigma, St. Louis, MO), ketamine (Ketalar, Pfizer, Turkey), and sodium valproate (Depakin, Sanofi, Turkey) were dissolved in saline, and carbamazepine (Novartis, Turkey) was suspended in 8.5% methylcellulose. Saline and drug solutions were given intraperitoneally (IP) in a volume of 4 ml/kg.

**Table 1:** Group names and treatments after 24 hr of fasting

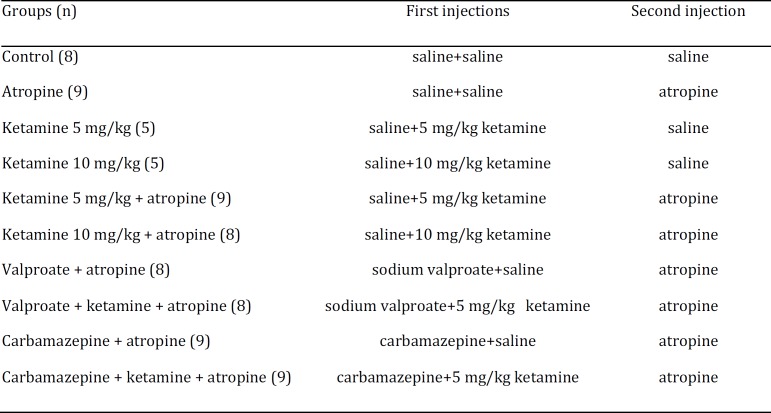

**Table 2:** Effect of ketamine and its combinations with valproate or carbamazepine on atropine-induced convulsions in fasted mice after food intake

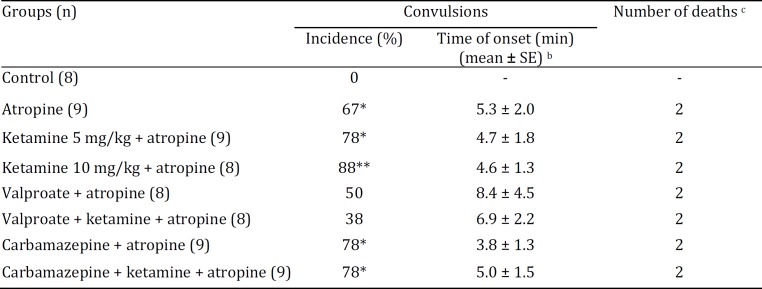

^b ^Calculated from seizing animals.

^c^ Caused by generalized convulsions (stage 5).

[*n*]: number of animals.

F(6,45) = 0.478; *P* = 0.821.

^*^ *P<*0.01, significantly different from control (saline) group.

^**^ *P<*0.001, significantly different from control (saline) group.

a Mice fasted for 24 hr were injected IP with saline, 5 or 10 mg/kg ketamine, 250 mg/kg sodium valproate, or 24 mg/kg carbamazepine pretreatments simultaneously with saline or 5 mg/kg ketamine 10 min prior to IP saline or atropine (2.4 mg/kg) treatments and were given free access to food 20 min later.

**Table 3:** Number of animals showing seizure stages in all groups

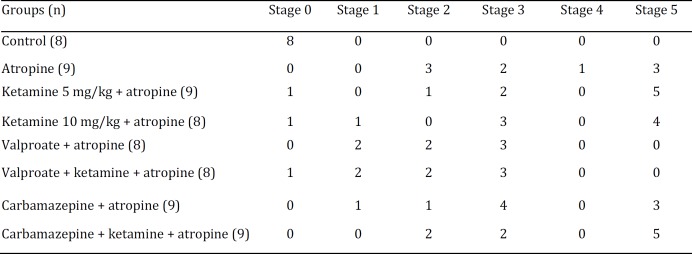

[*n*]: number of animals

***Evaluation of the effects of ketamine and its combinations with antiepileptics on convulsions***

After being weighed, mice were moved to clean cages with fresh bedding and were deprived of food with free access to water. Following 24 hr of fasting, the animals were reweighed and given firstly saline, 5 or 10 mg/kg ketamine, 250 mg/kg sodium valproate, or 24 mg/kg carbamazepine pretreatments simultaneously with saline or 5 mg/kg ketamine injections. Ten minutes later, they were treated with saline or 2.4 mg/kg atropine. Group names and the treatments were shown in Table 1. Soon after saline or atropine administrations, mice in the control, atropine, ketamine 5 mg/kg, ketamine 10 mg/kg, ketamine 5 mg/kg+atropine, ketamine 10 mg/kg+atropine, valproate+atropine, valproate+ketamine+atropine, carbamazepine +atropine, and carbamazepine+ ketamine+atropine groups were individually placed in wire mesh cages. Twenty minutes later, they were given food pellets and allowed to eat *ad libitum*. All mice were observed for 30 min for the incidence and onset of convulsions. Using a modified Racine’s scale (17), seizure activity was quantified by staging: (0) no difference; (1) freezing and gustatory movements; (2) forelimb clonus; (3) forelimb clonus with rearing; (4) forelimb clonus with rearing and falling down; and (5) generalized convulsions with rearing, falling down and jumping. A convulsive response was assessed as forelimb clonus with rearing. The onset of convulsions was defined as the time latency from starting to eat and appearance of first forelimb clonus with rearing. The incidence of convulsions was expressed as the percentage of animals displaying either stage 3, 4, or 5 activity in each group. The number of animals per group was 5–9.

Experiments were carried out between 09:00 and 15:00 hours in a temperature controlled (21±2 ^°^C) quiet room. Observers were blind to the treatments.

***Statistical analysis***

The onset of convulsions data was evaluated using one-way analysis of variance (ANOVA). Fisher’s exact test (*n*<20) was used for the evaluation of the frequency of the incidence of convulsions.

## Results

After fasting for 24 hr, the body weights of the animals fell to approximately 84–87% of the starting body weights. 

Table 2 shows that atropine treatment caused convulsions in fasted mice after food intake. The incidence of convulsions was statistically significant when compared with the control group (*P*<0.01). Ketamine, valproate, and carbamazepine pretreatments were ineffective in preventing the development of convulsions. When compared with the control group, the incidence of convulsions was significantly higher in the ketamine 5 mg/kg+atropine (*P*<0.01), ketamine 10 mg/kg+atropine (*P*<0.001), carbamazepine+atropine (*P*<0.01), and carbamazepine+ketamine+atropine (*P*<0.01) groups. In contrast, the incidence of convulsions in the valproate+atropine and valproate+ketamine+atropine groups did not differ significantly from the control group (*P*>0.05).

One-way ANOVA did not reveal main effect of treatment on onset of convulsions (F(6,45)= 0.478; *P*=0.821) (Table 2). Seizure stages were also indifferent between atropine and ketamine+atropine, carbamazepine+atropine, and carbamazepine+ ketamine+atropine groups (Table 3).

None of the animals in the ketamine 5 and ketamine 10 mg/kg groups developed convulsions (data not shown).

## Discussion

Fasting-induced decreases in B_max_ and K_d_ of the (^3^H)glutamate binding sites in the brain and suppression of the convulsions by the noncompetitive NMDA receptor antagonist MK-801 were evaluated as biochemical and behavioral evidence for a possible glutamatergic hyperactivity underlying seizures in fasted animals (3). Unfortunately, the present results obtained with another non-competitive NMDA antagonist ketamine do not appear to support this suggestion. The anticonvulsant efficacy of ketamine and MK-801 in experimental models of seizures has been attributed to NMDA receptor channel blockade (11, 18). Both of them are high-affinity open channel blockers in NMDA receptors. However, ketamine was found to be less potent than MK-801 in dissociation from the open channel (19) and antagonizing agonist-induced inward current responses in hippocampal neurons (20). Ketamine exerted less pronounced effect than MK-801 in blocking convulsions induced by glutamatergic agents N-methyl-DL-aspartic acid (21) and 4-aminopyridine (22) in mice and rats, respectively. These differences between ketamine and MK-801 may explain the ketamine’s ineffectiveness in suppressing seizures in fasted animals. Although ketamine’s primary site of action appears to be the NMDA receptors, the drug profoundly inhibits muscarinic signaling in Xenopus oocytes recombinantly expressed M_1_ muscarinic receptors (23), and increases both glutamate and dopamine concentrations in dialysate collected from the medial prefrontal cortex of rats (24). These mechanisms might counteract to a possible anticonvulsant effect of ketamine in the present seizures.

Ketamine produces its anticonvulsant effect at a dose range of 0.5–50 mg/kg in convulsions-induced by electrical stimulation (25) or by various chemical substances, such as NMDA (26), pilocarpine (27), lidocaine (28), 4-aminopyridine (22), bicuculline (29), pentylenetetrazole (30), and kainate (31). The doses used in the present study for ketamine, 5 and 10 mg/kg, were suggested to represent optimal doses for anticonvulsant activity. Nevertheless, seizures in fasted animals may require higher doses because of showing refractoriness to most antiepileptic treatments (5, 6). On the other hand, there are clinical and experimental observations for ketamine-induced convulsant or proconvulsant activity. Among those are the occurrence of seizures during anesthesia (32) or activation of epileptic foci (33) in patients and augmentation of pentylenetetrazole convulsions (34) or shortening of latency to sound-induced seizures (35) in animals. Ketamine did not produce any such activity at least in the dose range used in the present study.

Animal studies have shown that the combination of antiepileptic drugs with various agents could enhance the antiepileptic activity (36–38). In this respect, ketamine provided more effective treatments by potentiating the anticonvulsant effects of valproate and carbamazepine in seizures in rats and mice (12, 39). To investigate whether ketamine possesses similar efficacy in convulsions observed in fasted animal, the drug was combined with both antiepileptics. Carbamazepine at the dose of 24 mg/kg exerted prolongation of onset of convulsions, and sodium valproate at the dose of 250 mg/kg, partly suppressed the development of convulsions (5). Carbamazepine neither reduced the incidence nor delayed the onset of seizures. These findings are partly compatible with the previous findings. Ketamine coadministration did not provide an enhancement in carbamazepine’s activity. Lack of statistical significance in the incidence of convulsions in animals pretreated with valproate (50%) is consistent with the previous results (5, 40). Combination of valproate with ketamine produced a lower (38%), but also insignificant, incidence of convulsions compared with the atropine group (67%). The seizure stages in carbamazepine and valproate given animals were also unaffected by ketamine coadministration. Similar results were demonstrated when carbamazepine and lamotrigine were combined with the antidepressant amitriptyline (41).

## Conclusion

Ketamine exhibited lack of anticonvulsant efficacy when given alone and produced no synergistic effect when given with carbamazepine or valproate in convulsions developed after atropine treatment and food intake in 24 hr fasted mice. As for the assessment of the role of glutamate in these convulsions, new studies with different doses of ketamine or other NMDA antagonists are required.
